# The creation of an adaptable informed consent form for research purposes to overcome national and institutional bottlenecks in ethics review: experience from rare disease registries

**DOI:** 10.3389/fmed.2024.1384026

**Published:** 2024-04-17

**Authors:** Annalisa Landi, Yanis Mimouni, Viviana Giannuzzi, Franz Schaefer, Annagrazia Altavilla, Spencer Gibson, Daria Julkowska

**Affiliations:** ^1^Fondazione per la Ricerca Farmacologica Gianni Benzi Onlus, Bari, Italy; ^2^European Joint Programme on Rare Diseases Coordination, INSERM, Paris, France; ^3^Division of Pediatric Nephrology, Center for Pediatrics and Adolescent Medicine, University of Heidelberg, Heidelberg, Germany; ^4^Teddy European Network of Excellence for Paediatric Research, Pavia, Italy; ^5^Espace Ethique PACA-Corse Assistance Publique - Hôpitaux de Marseille (AP-HM), Hôpital de la Timone, Marseille, France; ^6^Department of Genetics and Genome Biology, University of Leicester, Leicester, United Kingdom

**Keywords:** informed consent, rare diseases, research, ethics review, registries

## Abstract

**Background:**

The lack of harmonization of evaluation criteria by Ethics Committees in the European Union (EU) has led to inconsistent ethics reviews received by research sites participating in multicenter non-interventional studies. The European General Data Protection Regulation (GDPR) appears to be implemented at national level with a substantial degree of variance in interpretation. The European Reference Networks (ERNs) were struggling in setting an Informed Consent Form (ICF) for registries, allowing reuse of data for research purposes. The aim of this work is to develop an adaptable ICF for research purposes to be used in ERN registries.

**Methods:**

To work on this challenge, a team was established within the European Joint Programme on Rare Diseases (EJP RD) to develop a patients’ registry ICF template allowing easy adaptation to ERNs, country, and site-level specificities. ERN and patients’ representatives validated the choice of developing a GDPR-compliant template for research purposes. The feedback received from 34 Ethics Committees on the Clinical Patient Management System ICF, including the submission of patients’ data to the ERN registries and the EU consent regulatory framework were analyzed along with existing ontologies for data access and reuse. An adaptable ICF was developed following iterative cycles of consultation and review by clinicians, research experts, ethics and regulatory advisors, and patients’ representatives. The development of pediatric material for minor participants was also undertaken.

**Results and Conclusion:**

Research oriented ICF templates for adults and for parents/legal representatives of patients were released in 26 national languages. This adaptable ICF aims to foster, according to patients’ preferences, the reuse of registries data for research purposes in compliance with the applicable laws and standards. Pediatric material is being finalized to collect minors’ assent. ICF machine-readability is also progressing to enhance data discovery and facilitate its access and reuse conditions.

## Introduction

In recent years, there is growing awareness and consensus in the Rare Diseases (RD) community about the need for secondary use of healthcare data for research purposes (1, 2). Systematic data collection, access and sharing practices are needed to foster RD research and many initiatives are ongoing to address these topics (3, 4).

Nevertheless, the use of health data in the RD field may be affected by the low prevalence of the diseases and the data collection heterogeneity (5). As health data reuse is being scaled up, there is an urgent need to reconcile the benefits of data access and sharing with privacy rights and constraints, as well as with ethical and regulatory requirements (6). The European General Data Protection Regulation (GDPR), Regulation (EU) 2016/679 (7), enables a new legal framework for data protection in Europe and provides different legal bases for processing personal data. Nevertheless, some issues might be triggered especially within the “research exception” option (8).

In RD research, also when involving children, it is crucial to ensure clear and explicit consent for data processing and its subsequent reuse (9). This necessitates, in the case of minors, not only obtaining the authorization of their parents/legal representatives but also seeking the assent of the minors themselves tailored to the unique needs of minors (10). This dual requirement of consent and assent aligns with various international laws and guidelines that emphasize the involvement of children in health-related decision-making.

Harmonized tools for the standardization of practices need to be further developed to help in the assessment procedures at national level when processing special categories of data (6).

While the use of personal data including health data is regulated around the world by data protection laws allowing citizens to control the use of their personal data, the large diversity of regulations and healthcare landscapes across and even within countries results in challenges for researchers in processing and sharing data in collaborative research activities (11).

In the field of RD research, multinational collaboration is often essential due to the limited prevalence of these conditions, which makes them a formidable challenge for any single country or region. Such research, distributed across various laboratories and clinics worldwide (12), faces the complexity of diverse ethical standards and procedures. This diversity arises also because each Ethics Committee may operate under its own set of rules and require different documents and contents for approval. This lack of harmonization in ethical evaluations across the European Union (EU) can lead to inconsistent ethical reviews for the same study at different research sites (13–15). The new European Clinical Trials Regulation introduces a streamlined process, allowing for a single study submission and review by one designated Ethics Committee per country (16). Nevertheless, this change, aimed at simplifying the review process, unfortunately, does not extend to observational studies.

Furthermore, as anticipated above, the advent of the GDPR (7) also has not solved this fragmentation since it is implemented at the national and local Ethics Committee levels with a certain degree of variance in interpretation. Thus, additional legislative efforts are required to guarantee comparable ethical standards among sites (17). For all these reasons, non-interventional multicenter research projects face challenges to comply with the applicable requirements and to obtain approval from all competent Ethics Committees involved.

In the RD field, the European Reference Networks (ERNs) have struggled in setting an Informed Consent Form (ICF) for registries that is acceptable to Ethics Committees across the EU. A generic ICF originally developed for the Clinical Patient Management System (18), an online platform for transnational clinical consultations among ERN Members which contained a section on data handling in registries, was not accepted by all Ethics Committees to which it was submitted. Moreover, age-appropriate information for pediatric participants and/or assent forms were not developed by most ERNs, since the assent is not a legally mandated requirement but requested only by individual Ethics Committees according to local provisions. In the framework of the European Joint Programme on Rare Diseases (EJP RD), an Informed Consent Facilitation Group was established to support the ERNs addressing these challenges. The Group was composed of members of the EJP RD Advisory Regulatory and Ethics Board, the independent Ethics Advisor team, the EJP RD Coordination team, clinical experts from the ERNs, and experts in pediatrics. All the authors of this manuscript are part of the Informed Consent Facilitation Group.

The group examined the current practices and regulations surrounding informed consent, incorporating insights from the feedback provided by various Ethics Committees, with the aim to develop an adaptable ICF for research purposes to be used for ERN registries. Key ethics and data protection challenges encountered during ethics review processes were identified during the review. To address these challenges, a harmonized framework for informed consent was developed. This framework is uniquely designed to be adaptable, allowing for necessary modifications to fit national and local requirements.

## Materials and methods

### Analysis of the Clinical Patient Management System ICF submitted to the Ethics Committees

**FIGURE 1:**
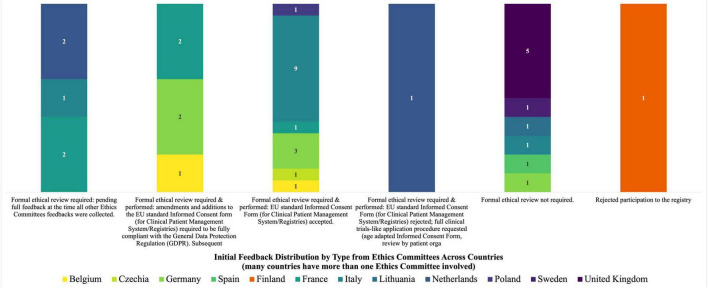
Initial feedback distribution by type from ethics committees across countries.

The initial feedback received following the submission of the Clinical Patient Management System ICF to 38 concerned Ethics Committees is shown in Figure 1. To note, for many countries more sites were involved, and so more than one Ethics Committee was concerned in the revision process.

The Group performed an in-depth reading and analysis of the initial ICF developed for the Clinical Patient Management System to process patients’ registry data for research purposes, the opinion letters that the European Rare Kidney Disease Reference Network members received from 34 out of the 38 Ethics Committees to which the ICF was submitted, the amended ICFs that followed the implementation of Ethics Committees’ comments as well as the other registry ICFs developed by individual ERNs. It was highlighted that within the same country different feedback was found for the same ICF. Moreover, the EU rules (including GDPR), guidelines and standards applicable to the consent were detected and consulted (7, 10, 16, 19–24).

Finally, an analysis of the compliance of the ICF with the GDPR was performed to identify the information to be provided to data subjects, as listed in Article 13.

### Development of the adaptable ICF template

A meeting with ERN representatives was scheduled on 15 December 2020 to discuss two alternative approaches: (1) to develop a new version of the ICF focused on fully GDPR compliant reuse of ERN registry clinical data for research purposes and allowing adaptations at different levels, or (2) to create a dual ICF covering both the primary and secondary use of the ERN registry clinical data. Based on the consultation, the first approach was agreed.

**FIGURE 2:**
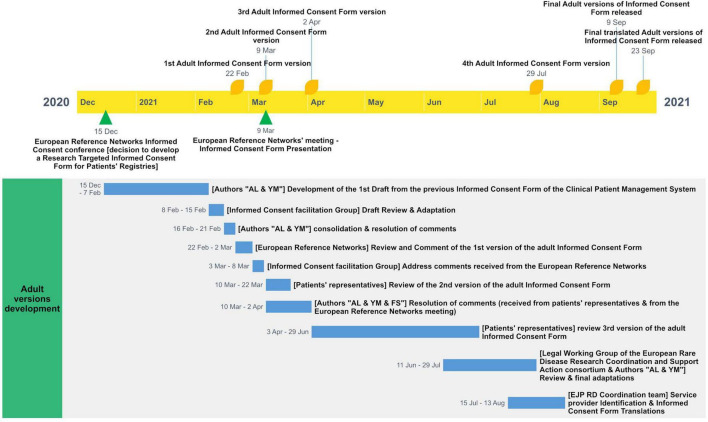
Timeline for the development of the adult version of the ICF template.

A first draft of the adaptable ICF template was developed starting from the previous version of the Clinical Patient Management System ICF. Then, the draft went through several rounds of revisions and adaptations by various experts. Iterative cycles of consultation and review by ERN clinicians and researchers, ethics and regulatory advisors, and patients’ representatives, including Young Persons Advisory Groups (YPAGs) were undertaken. Around 34 experts provided their contribution during the cycles of consultation. The comments were addressed by the Group and the updated ICF versions were consolidated until their finalization. Figure 2 shows the different steps and the timeline for the development of the adult version of the ICF template.

The template aimed to give subjects the chance to consent to the participation in the registry while providing them additional choices related to the reuse of their data. Both EJP RD and external experts revised the ICF before finalization and translation from English to 26 other European languages.

### Development of the pediatric material for minors

Separate material for pediatric participants (i.e., assent forms and informative material for minors) was developed only by few ERN registries. Only one out of 38 Ethics Committees reviewing the general ICF requested age-appropriate material for minors. Despite this, the Informed Consent Facilitation Group recognized the importance of developing pediatric material for minor participants in the ERN registries and started with the development of material for adolescents. This development process was guided by a participatory methodology, adhering to data protection norms and children’s rights, and was influenced by the principles outlined in the draft guide by the Council of Europe (25).

Starting from the adaptable ICF for adults, along with existing materials from ERNs, including assent forms and informational content for minors, an assent form template specifically for adolescents aged 12–17 was created during three Pediatric Expert Patients Training Courses, the first of which consisted of five sessions, organized under the EJP RD. This approach ensured that the information provided is concise, transparent, and understandable, presented in a format accessible to adolescents, and utilizes clear, straightforward language.

During the training courses, attended by approximately 26 pediatric patients, participants completed a questionnaire to assess their understanding of the objectives of patients’ registries, the relevance of children’s rights in data protection, and the GDPR rules. They were given an overview of children’s rights and data protection rules. Emphasis was placed on the concept of the “right to an open future.” This principle posits that children should be shielded from making certain irreversible decisions, ensuring that their future options remain open until they reach adulthood and can make informed choices. This concept is especially pertinent in the context of research involving personal health data in rare diseases, where the implications of data use can significantly impact a child’s future.

Participants were given ICF templates designed for adults and instructed to use a color-coding system for their feedback: terms difficult to understand were to be marked in red; clear information deemed relevant for giving consent or assent was to be highlighted in green; and parts deemed understandable, irrelevant, better placed elsewhere, or that needed rephrasing, were to be highlighted in pink. Additionally, participants were encouraged to provide open-ended feedback and to suggest improvements, e.g., information that could be better explained through visual aids. This interactive approach aimed to refine the consent forms to be as clear and relevant as possible.

Based on the course outcomes, a draft version of the assent form for adolescents was created and shared with the participants for review as well as a glossary. Concurrently, similar revision processes were undertaken with some existing YPAGs to further validate the assent form. Finally, the creation of informative material and assent form for children was also deemed necessary and planned as a next step.

**FIGURE 3:**
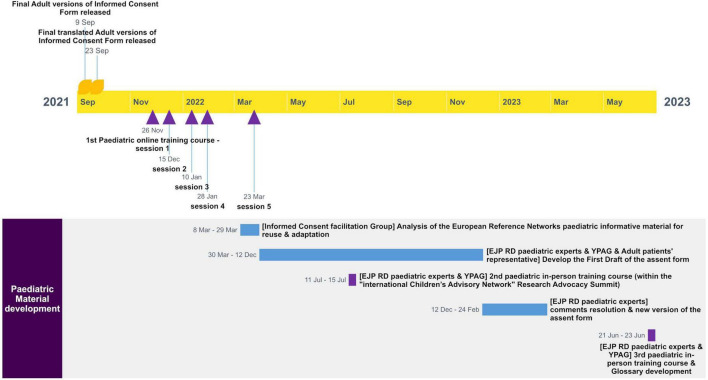
Timeline for the development of the pediatric material.

Figure 3 shows the timeline for the development of the pediatric material.

### Scouting of existing ontologies

The terms of the Data Use Ontology (26), the Informed Consent Ontology (27), and the Automatable Discovery and Access Matrix (28) were assessed to see if they could be used to adequately encode the consent and data access and reuse conditions defined in the adaptable ICF. Such an encoding would greatly assist with the digitalization of a participants consent in a potentially interoperable format (i.e., that can be used by various Information Technology systems). The process consisted of aligning the terms and codes from these ontologies and matrix with corresponding terms and phrases in the ICF. The outcomes of this initial investigation were then shared with ontology experts and Information Technology developers from the EJP RD, followed by proposing ways to incorporate these alignments into data models and tools, to enable a machine-readable ICF. As an additional test of functionality of the proposed alignment, we established various “consent profiles,” based on patients’ preferences expressed in the consent form and attempted to represent them in a machine-readable format.

## Results

### Analysis of the Clinical Patient Management System ICF submitted to the Ethics Committees

Thirty-four out of the initial 38 Ethics Committees, from 12 countries, provided a wide range of responses at the time of the analysis following the submission of the Clinical Patient Management System ICF for approving the reuse of patients’ registry data for research purposes and after the preliminary feedback shown in Figure 1: 10 stated that ethics approval was not required for this type of activity, 17 accepted the Clinical Patient Management System ICF after formal ethical review without any changes, 6 accepted an amended version of the ICF following the inclusion of additional information that made it fully compliant with the GDPR, and 1 requested a full application procedure according to the clinical trials requirements, including the provision of informative materials and assent form for minors with compulsory review by patients’ organizations. The documents produced for the latter Ethics Committee were also used by two other sites in the same country (Netherlands). Feedback from three other Ethics Committees was still pending at the time of analysis while one site rejected its participation in the registry.

Most modifications requested by the Ethics Committees were related to compliance with GDPR and in particular: information relating to the duration of the data storage; the (geographical) location of the registry; the data subjects’ rights; the name and contact details of the data controller and the Data Protection Officer (DPO); and the reference to the supervisory authority to exercise data subjects’ rights.

The missing information identified during the analysis and possible improvements of the existing form were discussed with the Informed Consent Facilitation Group members, with EJP RD data experts and ERN representatives.

### Development of the adaptable ICF template

The adaptable ICF template was designed specifically for research purposes. It addresses the collection, access, storage, and potential future (re)use of data within the ERN registries, but it does not cover the primary use of health data in the Clinical Patient Management System. The structure of the template includes a primary consent section for data inclusion in the ERN registries for specific research objectives. In addition to this primary consent, participants can select five optional consent choices according to their preferences: use of data to support commercial projects; transfer of data to non-EU countries; linking of data to existing databases/registries; possibility for the patient to be recontacted for any research project or clinical study; will to be informed about any incidental finding. These optional consent choices were chosen by the Group and agreed with the experts involved during the consultation phases considering the main reasons for which participants might refuse to participate in the registry (e.g., in case of data transfer outside the EU countries) and choices that might be not applicable for all ERNs (e.g., incidental findings only for ERNs handling genetic data).

Importantly, these optional consents are not a prerequisite for participation in the ERN registries: they are designed to provide participants with a greater control over their data and to express their preferences.

Two versions of the ICF were developed. One for adult patients, the other for parents or legally designated representatives of minors or incapacitated individuals.

To enhance the flexibility and applicability of the ICF across different contexts, the template was designed with modifiable sections. These adaptable areas were clearly marked to facilitate easy customization. This allows to tailor the ICF to the specific requirements of individual ERNs or sites, e.g., to describe the disease(s), indicate the type of collected data and the possible link with other registries or databases, provide information on where and for how much time the data is stored, how the research results exploiting the registry will be communicated, and the contact details of the reference person or entity to be contacted for inquiries, including information on the data controller and the DPO. The adaptable areas allow also to comply with any relevant national and local laws and with site specificities (e.g., information on insurance). This approach ensures that the ICF can be effectively used in diverse settings while adhering to varying legal and regulatory standards.

The final versions of the adaptable ICF templates were released, following different rounds of consultations, with translations into 26 European national languages and made publicly available on the EJP RD website (29).

### Development of the pediatric material for minors

The first pediatric workshop revealed a general lack of awareness of children and adolescents about the objectives, contents, procedures and uses of patient registries. Most of them also declared not to be aware of children’s rights and data protection principles. Those who declared to be aware of these issues were trained within YPAGs, patients or consultative groups.

Children participants provided suggestions to enrich the developed assent form for adolescents by including (1) a lay glossary explaining some concepts (e.g., data controller, DPO, data protection authority, and commercial use), (2) child-friendly interactive elements and diagrams, and (3) flowcharts or images explaining the flow of personal data in the registry. Moreover, they recommended to post on the registry website more comprehensive child-friendly information about the adopted safeguards and data protection policies, and to post lay summaries with results of the studies carried out with the data of pediatric patients participating in the registries. They also underlined that children and adolescents must be protected against being engaged in certain irreversible choices. In this perspective, the question of the commercial use of data was raised and it was underlined the need to require an opinion from an Ethics Committee on a case-by-case basis, considering the best interests of the child.

The need to clearly differentiate research conducted with commercial sponsorship, as outlined in the adaptable ICF, from academic studies was highlighted from experts. In particular, it was discussed that no financial benefit is foreseen for data subjects and data could become property of the concerned company that could also be used for further commercial purposes and for patents.

Within the second EJP RD pediatric training workshop, the assent form developed for adolescents was submitted to further consultation and conclusions were further discussed with pediatric participants. In the third training workshop, a child friendly glossary was developed.

At the time of this manuscript submission, the assent form template for adolescents is in the process of final consultation before the release of its final version and the preparation of informative material and assent form for children considered as further step.

### Scouting of existing ontologies

The review of informed consent and data utilization frameworks examined ontologies and engaged in experts’ consultation resulting in the identification of 67 different codes. These codes corresponded to specific terms or expressions found within the ICF: 44 of these codes belonged to the Informed Consent Ontology, 22 to the Data Use Ontology and one code pertained to the Disease Ontology, specifically referring to the disease under study. The identified codes from the Data Use Ontology and the Informed Consent Ontology were grouped into several thematic categories. The first category, the Data Use and Sharing Permissions, encompassed terms related to the permissions for using and sharing data. These terms define specific conditions under which data may be accessed and used, particularly in research contexts. The terms of the second category, Consent and Legal Compliance, relate to the informed consent processes, legal obligations, and ethical compliance in data collection and use. The third Patient Engagement and Communication category includes terms emphasizing the importance of patient engagement, understanding the consent forms, and facilitating the effective communication regarding data use. Finally, the terms of the Data Management and Security category deal with data management practices and security measures to protect data integrity and confidentiality. Each category represents a different aspect of data handling in medical research, encompassing ethical considerations, legal compliance, patient interaction, and data protection.

The undertaking of this ICF mapping exercise contributed to the refinement of the Data Use Ontology through the elimination of subclasses that caused ambiguity for the users and the addition of a new optional consent category in the ICF (30). This enhancement enabled the inclusion of provisions for the reuse of data in commercial projects (e.g., industry sponsored drug development trials) and the disclosure of incidental finding.

Ultimately, this analysis facilitated the creation of 32 unique “consent profiles” using the DUC profile creator developed by EJP RD (31) that exploits the Common Conditions of Use Elements (32). This reflects the incorporation of five optional consent options within the ICF templates, thereby augmenting the granularity and flexibility of consent documentation and data reuse in scientific research settings.

## Discussion

The adaptable ICF template released in the framework of EJP RD aims to foster the reuse of registry data for research purposes in compliance with the applicable relevant laws and standards as well as patient preferences.

The relevance of this work consists, on one hand, in giving subjects the chance to consent to the main purposes of the registry while providing them with the choice about other data processing activities (e.g., the transfer of data to non-EU countries, the reuse of data for research projects with commercial sponsors or the possibility to be re-contacted to participate in other research projects). It aims to reinforce the concept of “granularity,” as stated in the GDPR and to create personalized “consent profiles” based on patients’ preferences on their data use. On the other hand, the concept of avoiding a “one-size-fits-all” approach for the ICF was addressed. The focus was on developing an adaptable ICF that could conform to diverse national and local regulations and standards, allowing for straightforward text modifications. This concept was further expanded to the assent template, generated based on the recommendations from the young patient. The involvement of children in developing pediatric assent and their feedback highlighted the importance of the participatory methodology to take decisions and implement practices adequate to children needs, expectancies and rights. A greater involvement of children in developing specific child friendly tools in rare diseases should be promoted.

It has been demonstrated that RD patients and parents are keen to make their samples and data available to researchers if this is done with respect and reciprocity (2, 11, 33, 34).

We strongly believe that our tool could increase their willingness to share data and foster their active participation in the RD registries. Moreover, pediatric material has been developed to collect minors’ assent to be informed and agree on the use of their data. Considering their apparent lack of awareness of registries as research tools, more efforts and education campaigns will be needed to inform pediatric patients about the value of RD registries and about their rights in the context of data use and protection.

Another effort is currently making the adaptable ICF machine-readable, leveraging on the ontology mapping and the created consent profiles. This work exploits the EJP RD created Data Use Condition tool (31) using the Common Conditions of Use Elements, that were partly derived from the development of this ICF, and on the use of the Open Digital Rights Language ontology (35) for semantic implementation. Enabling machine-readability of access and reuse conditions also considers ERN registry data access policy, data sharing agreement and data transfer agreement, jointly developed by EJP RD and the European Rare Disease Research Coordination and Support Action consortium (36). We aim through these approaches to enhance the ERN registries data discovery and display the access and reuse conditions when querying for RD information, and to facilitate the data submissions and access requests for researchers and data access committees following the successful implementation of the Data Use Oversight System (37) and the BBMRI Negotiator (38). We are also considering to eventually enable patients to use the ERN registries’ websites for exercising their rights. This includes developing age-appropriate information and tools, dynamically modifying consent, accessing their data, and having the ability to update their data. This goal is currently still a topic of discussion and has not been finalized.

Our research highlights the importance of assembling a multidisciplinary team with diverse and complementary expertise when establishing and managing patient registries, i.e., experts in data management, regulatory compliance, ethics, and legal matters, along with patients’ representatives. One crucial finding from our work emphasizes the need for the technical development of these registries to incorporate data usage conditions within a tailored data governance framework, since the design phase.

Ensuring that RD registries meet high-quality standards in technical infrastructure, ethics, and data protection is imperative. To address potential risks, specific safeguard measures, such as conducting a Data Protection Impact Assessment under Article 35 of GDPR, should be implemented. This is particularly vital when dealing with vulnerable individuals, like children.

An unresolved question relates to the validity of informed consent regarding data reuse, especially in the context of the European Commission’s proposal for a European Health Data Space (39). This complexity is further compounded when considering the integration of ERN registries into this framework, because it raises intricate legal considerations surrounding data processing.

The outcomes of this work not only aim to promote harmonized practices and facilitate the secondary use of health data in general, but also provide adaptable templates. Furthermore, these outcomes can assist the Registry Data Access Committees by offering a more transparent view of patients’ preferences regarding the use of their data.

## Conclusion

In conclusion, this work represents a significant milestone that serves as a model in various research activities dependent on consent. It is highly valuable for the RD community and holds potential for extension and application to other disease communities. It might also be used to complement the information on informed consent included in the European Medicines Agency Guideline on registry-based studies (40). Additionally, the ICF developed here is currently being tested by other registries and research projects. Its successful implementation is expected to enable the secondary use of healthcare data in various other research endeavors, including initiatives like the European Rare Diseases Research Alliance (41); thereby broadening the scope and impact of this work.

## Data availability statement

The original contributions presented in this study are included in this article/supplementary material. Further inquiries on the original data can be directed to the corresponding author.

## Author contributions

AL: Conceptualization, Formal analysis, Methodology, Writing – original draft, Writing – review & editing. YM: Conceptualization, Formal analysis, Methodology, Writing – review & editing. VG: Methodology, Writing – original draft, Writing – review & editing. FS: Conceptualization, Methodology, Supervision, Writing – review & editing. AA: Methodology, Writing – review & editing. SG: Formal analysis, Methodology, Writing – review & editing. DJ: Conceptualization, Methodology, Writing – review & editing.
